# Stomatal pore width and area measurements in *Zea mays*

**DOI:** 10.17912/micropub.biology.000893

**Published:** 2023-08-04

**Authors:** Jessica R Lucas, Brittany Dupree

**Affiliations:** 1 Biology, University of Wisconsin - Oshkosh, Oshkosh, Wisconsin, United States of America

## Abstract

Stomatal pores are adjustable microscopic holes on the surface of photosynthetic tissues that help regulate multiple aspects of plant physiology. Stomatal pores facilitate gas exchange necessary for photosynthesis, water transport, and temperature regulation. Pore size is influenced by many intertwined environmental, molecular, cellular, and physiological cues. Accurate and precise measurements of pore size is important for understanding the mechanisms that adjust pores and plant physiology. Here we investigate whether conventional pore measurements of width are appropriate for the economically important crop plant *Zea mays* . Our studies demonstrate that pore area is a more sensitive measurement than width in this plant.

**Figure 1. Zea mays Stomatal Pore Width and Area.**
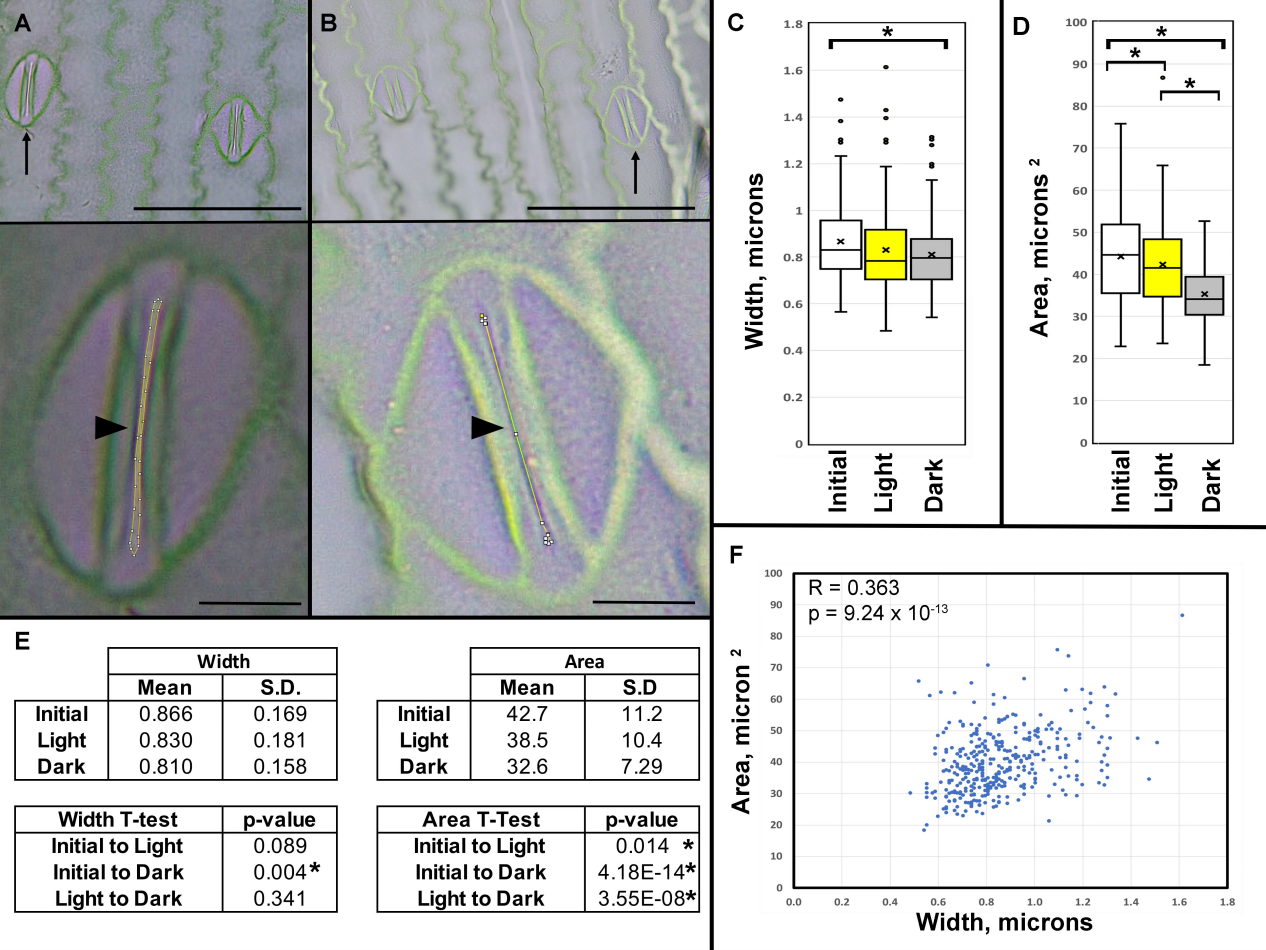
(A) Open stomata and surrounding epidermal cells exposed to light. (B) Closed stomata after two hours of darkness. In upper panels, arrows indicated the stomata enlarged in lower panels. In lower panels, arrowheads illustrate where pore width was measured and dotted lines mark the pore area measurement. Mag bars represent 5 microns in top panels and 1 micron in lower panels. (C) Width of stomatal pores before treatment (initial), after two hours of light (light), and after two hours of darkness (dark) (N= 418 stomates, 12 leaves from 12 different plants). Bracket and asterisk (*) denoted p=0.004 from Student’s T-test. (D) Area of stomatal pores measured from initial, light, and dark treated leaves. Asterisks signify that p-values of all paired T-tests were less than 0.02. (E) Quantification of 418 stomatal pore widths and areas, and statistical analyses to evaluate differences between treatments. Asterisks indicate significant results. (F) Pearson’s Correlation analysis between pore width and area measurements from 418 stomata.

## Description

**Description**

Stomata are essential anatomical components in most land plants that regulate gas exchange in photosynthetic tissues (Gupta *et al* ., 2020; Buckley 2019; Lee and Bergmann 2019). Each stomate is composed of two guard cells that surround an adjustable opening in the epidermal surface called the aperture or pore, and each leaf can contain hundreds of stomata (Bergmann and Sack 2007; Lucas *et al* ., 2006). Open stomatal pores allow the free diffusion of water vapor, carbon dioxide, and oxygen between the internal plant tissue and the surrounding air (Lin *et al* ., 2022; Wang *et al* ., 2022). In *Zea mays* and many plants, stomatal pores open in response to light to facilitate gas exchange needed for photosynthesis, respiration, and water movement via evapotranspiration (Peng *et al* ., 2022; Wang and Chen 2020). Pores close to preserve water and in the dark of night (Peng *et al* ., 2022). Drought stresses plant life because stomatal pores must close to retain water, which then prohibits gas exchange needed for photosynthesis (Hsu *et al* ., 2021; Gupta *et al* ., 2020; Kirschbaum 2004). Multiple adaptations and molecular pathways exist to maximize photosynthesis and maintain water homeostasis (Peng *et al* ., 2022; Rodrigues and Shan, 2022; Hsu *et al* ., 2021). Therefore, much research is devoted to elucidating the complex signaling mechanisms that integrate environmental and physiological cues that influence stomata, and pore size is an important experimental output (Wang *et al* ., 2022; Hsu *et al* ., 2021).

Quantitatively analysis of stomatal pore size requires skill and is labor-intensive (Millstead *et al* ., 2020; Jayakody *et al* ., 2017). Automated systems to measure pores have been recently developed, and these require propriety software and additional tissue preparations (Takagi *et al* ., 2023; Liang *et al* ., 2022; Millstead *et al* ., 2020; Li *et al* ., 2019; Jayakody *et al* ., 2017). Stomatal pore size is often experimentally determined by measuring the largest width of the pore from light microscope images using freely available FiJi software (Sai *et al* ., 2023; Jayakody *et al* ., 2017). These width measurements are a reasonable proxy for pore size when guard cells are reniform (kidney-bean shaped) and the aperture is a symmetrical ellipse (Takagi *et al* ., 2023). Reniform stomata are common in many plants including ferns, gymnosperms, dicotyledonous flowing plants and trees, and model plants Arabidopsis thaliana, Glycine max (soybean), and Vitus vinerfa (grape). However, stomatal pores in grasses and cereal crops are irregularly shaped, elongated, and flanked by “dumbbell” shaped guard cells ( Figure 1B ) (Nunes *et al* ., 2020; Wang and Chen 2020). Cereal crops and model species which have irregular pores include *Zea mays* (corn), *Triticum vulgaris* (wheat), and *Oryza sativa* (rice) and *Brachypodium distachyon* (purple false broom) (Hepworth *et al* ., 2018). Width measurements of grass stomatal pores may not accurately represent pore size (Liang *et al* ., 2023). Effective methods to study stomatal pores in agriculturally relevant grasses and crop plants are needed to help study and mediate damage to crop yields caused by increasing global temperatures and reductions in freshwater availability (Liang *et al* ., 2023). Measurements of pore area may better represent the size of the stomatal pore.

We investigated potential relationships and correlations between width and area measurements of *Zea mays* stomatal pores. We collected bright-field microscopy images of stomatal pores from *Zea mays* plants grown in a greenhouse. Pore width and area was measured from digital images using FiJi software using the line or polygon tool, respectively (Schindelin *et al* ., 2012). To ensure that our analyses included a range of pore sizes, we treated the initial greenhouse leaf samples with additional bright light from a growth light or darkness a light-tight drawer for two hours to open and close pores, respectively.

Stomatal pores responded to experimental light and darkness treatments. Stomatal pores were primarily open on both the initial leaf greenhouse samples and those exposed to laboratory light ( Figure 1A -D). Stomatal pore width and areas measured 0.866 mm (S.D. 0.169, N= 418) and 42.7 mm ^2^ (S.D. 11.2, N = 418) respectively on average before laboratory treatments ( Figure 1E ). Pore width and areas both slightly decreased after exposure to bright light and measured 0.830 mm (S.D. 0.181) and 38.5 mm ^2^ (S.D. 10.4) on average ( Figure 1E ). This decrease was anticipated as environmental conditions in the greenhouse and lab were not identical. As predicted, dark-treated stomatal pores were smallest on average, and width and area measured 0.810 mm (S.D. 0.158) and 32.7 mm ^2^ (S.D. 7.29) ( Figure 1E ). As expected, these mean data indicated that width and area measurements both reflected that *Zea mays* stomata responded to environmental conditions, and pores were smallest in darkness in comparison to leaves exposed to light.

Statistically significant differences emerged in area measurements among all three treatments ( Figure 1D -E). Width comparisons of the initial greenhouse and dark-treated stomatal pores indicated significant differences ( Figure 1C, E ). While width measurements showed anticipated trends, paired Student T-tests of width measurements did not meet the threshold for significance in all treatments. For instance, p-values for width comparisons between initial greenhouse and light-treated pores and light- and dark-treated pores were both above 0.05 ( Figure 1E ). Overall, area recordings captured and statistically demonstrated the observed and expected differences in *Zea mays* pore size among the three leaf treatments. Next, we explored whether pore width and area measurements correlated. Pearson correlation analysis indicated that *Zea mays* pore width measurements poorly correlated with area (R=0.363, p= 9.24 x 10 ^-13^ , Figure 1F ).

Overall, this study indicated that area measurements of *Zea mays* stomatal pores demonstrated statistical differences in different environmental conditions while width measurements did not. While average pore width measurements showed the expected phenotype (smaller pores in dark-treated leaves in comparison to greenhouse leaves), changes between other treatment conditions did not yield significant differences in width measurements. While manually collecting area data from digital images is more time consuming than width data in FiJi, these data collectively suggest area calculations appear more precise *Zea mays* and can detect small differences. As stomatal pores are finely adjusted to balance photosynthetic gas exchange and water loss, small differences in pore size may have larger impacts on plant physiology (Buckley 2019) . Functional genetic and agricultural experiments may not show dramatic differences in stomatal pore size between genotypes and conditions; therefore, precise measurement techniques that can capture small differences in pore size are needed (Liang *et al* ., 2023).

## Methods

**Methods**

*Zea mays* plants were grown from seed (Early Golden Bant from Urban Farmer, Lot #J7706) in a greenhouse in Oshkosh, Wisconsin June and July of 2022 with 15 hours of ambient sunlight. Greenhouse air temperatures varied diurnally between 70-95 ^o^ F. The fifth leaf from 4 to 6-week-old plants were used in each experiment, and stomatal from the adaxial side were examined.

In each experimental replicate, we excised three 2cm ^2^ adjacent leaf samples each the same leaf (10-14 cm from the ligule) with a razor blade and two plants were sampled for each replicated. Two leaf samples were processed for imaging immediately, two others were treated for two hours with light from a PAR38 Philips growth light bulb, and the remaining two samples were stored in a light-tight drawer. Excised leaf samples incubated in the light and dark within 5cm glass Petri dishes with buffer solution (10mM MES, 20 μM CaCl _2,_ and 50 mM KCl, pH6.15 with KOH) (Rui and Anderson 2016) . These experiments were repeated four distinct times using unique plants each time.

Images were collected using a Leica DM500 BF bright field compound microscope with integrated ICC50 digital microscope and Plan 40x objective lens (N.A. 0.65). As the white transmitted light from the microscope can trigger stomatal pores to open, clear nail polish (Sally Henson) replicas were made from each leaf sample and then imaged on the compound microscopy. Replicas were made by allowing a thin layer of nail polish to dry for 15 minutes on the abaxial side of leaf sections. The replicas were removed using forceps and then transferred to a microscope slide. Leaf samples incubated in buffer solutions were gently blotted dry with a Kimwipe before applying nail polish.

Stomatal pore width and area was quantified in FiJi and analyzed in Microsoft Excel. Area was determined using the polygon tool to trace the pore in FiJi. Pore width was measured with the line tool bisecting the pore (lower panels of Figure 1A and B demonstrate measurements ). Differences between treatment conditions were analyzed using paired Student T-tests. Pore width and area measurements from individual stomata were evaluated using a Pearson’s Correlation calculation.
